# RNA-Seq profiling of circular RNAs in human colorectal Cancer liver metastasis and the potential biomarkers

**DOI:** 10.1186/s12943-018-0932-8

**Published:** 2019-01-10

**Authors:** Hanchen Xu, Chunyan Wang, Haiyan Song, Yangxian Xu, Guang Ji

**Affiliations:** 1 Institute of Digestive Diseases, Longhua Hospital, Shanghai University of Traditional Chinese Medicine, Shanghai, 200032 China; 2 Department of General Surgery, Longhua Hospital, Shanghai University of Traditional Chinese Medicine, Shanghai, 200032 China

**Keywords:** Colorectal cancer liver metastasis, Circular RNAs (circRNAs), RNA-sequencing (RNA-seq), Biomarker

## Abstract

In this study, the secondary sequencing was used to profile circRNA expression in the tissue samples from three CRC patients with liver metastasis and three matched CRC patients. After verified some candidates in another 40 CRC and CRC-m samples by qRT-PCR, we further demonstrated that circRNA_0001178 and circRNA_0000826 were significantly upregulated in CRC-m tissues, and both of them had the potential for diagnosing liver metastases from colorectal cancer. Finally, the networks of circRNA-miRNA-mRNA base on these two circRNAs were constructed respectively. This study showed that differentially expressed circRNAs were existed between the tissue samples from colorectal cancer patients with and without liver metastasis. And also suggested that circRNA_0001178 and circRNA_0000826 may serve as a potential diagnostic biomarker for liver metastases from colorectal cancer.

**Electronic supplementary material:**

The online version of this article (10.1186/s12943-018-0932-8) contains supplementary material, which is available to authorized users.

## Main text

Circular RNAs (circRNAs) are an important member of the non-coding RNA family following microRNAs and lncRNAs. They are characterized by the absence of covalently closed loop structures at the 3′ and 5′ ends. Base on this closed structure, circRNAs are highly stable and not easily degraded [1]. As this mechanism of circRNA as a ceRNA is revealed, this is considered to be an important way for circRNA to play a role in disease progression. CircRNA can act as a sponge to adsorb miRNA, and then regulate the expression of miRNA and its target genes [2, 3]. In addition, circRNAs have a certain specificity in different tissues and are therefore suitable as biomarkers for cancers. Recent studies have shown that circRNAs are abnormally expressed in tumor tissues, and some circRNAs as the potential biomarkers have been discovered [4–6]. However, as far as we know, there is no study on the role of circRNAs in liver metastasis from colorectal cancer. In this study, we used the secondary sequencing technology to compare the expression profile of circRNAs in the tissue samples from colorectal cancer patients and colorectal cancer patients with liver metastasis. Then, we expand the sample size to verify some candidates and assess the diagnostic value of the circRNAs as the biomarkers for liver metastases from colorectal cancer. Finally, we constructed a network map based on two circRNAs and their respective potential binding miRNA-mRNAs to better investigate their potential mechanisms of action in colorectal cancer liver metastasis.

## Collection the tissue specimens

A total of 40 patients were involved in this study, including 24 colorectal cancer patients (CRC) and another 16 colorectal cancer patients with liver metastases (CRC-m). Tumor tissue samples of these patients obtained from the surgical treatment at the Department of General Surgery, Longhua Hospital affiliated to Shanghai University of Traditional Chinese Medicine, China. The samples were snap froze in liquid nitrogen after separated from the human body immediately and stored at − 80 °C before using. This study was approved by the Ethics Committee of Longhua Hospital, and the informed consent form was signed by every participant.

## RNA-seq analysis

The total RNA was isolated from the tissue samples using TRIzol reagent (Life Technologies, Carlsbad, CA) according to the manufacturer’s instructions. RNA integrity and DNA contamination were assessed using electrophoresis on a denaturing agarose gel. Before constructing the RNA-seq libraries, the Ribo-Zero rRNA Removal Kit (Illumina,San Diego, CA, USA) and the CircRNA Enrichment Kit (Cloud-seq,USA) were used to remove the rRNA and enrich the circRNAs. The RNA-seq libraries were constructed by using pretreated RNAs with TruSeq Stranded Total RNA Library Prep Kit (Illumina, San Diego, CA, USA) according to the manufacturer’s instructions. The libraries were denatured as single-stranded DNA molecules, captured on Illumina flow cells, amplified in situ as clusters and finally sequenced for 150 cycles on Illumina HiSeq™ 4000 Sequencer (Illumina, San Diego, CA, USA)according to the manufacturer’s instructions.

## Identification and quantification of human circRNAs

Paired-end reads were harvested from Illumina HiSeq 4000 sequencer, and were quality controlled by Q30. The reads were aligned to the reference genome/transcriptome with STAR software and circRNAs were detected and annotated with DCC software. CircBase database and circ2Trait disease database were used to annotated the identified circRNAs. Differentially expressed circRNAs were identified by T test between two groups.

## Validation of candidate circRNAs using quantitative real-time reverse-transcription polymerase chain reaction (qRT-PCR)

Total RNA was extracted from the tissue samples using TRIzol reagent (Life Technologies, Carlsbad, CA) and then reverse-transcribed into cDNA using the SuperScript First-Strand Synthesis System (Invitrogen, Carlsbad, CA, USA). The cDNA was used for qPCR using SYBR Green PCR Master Mix (Applied Biosystems, Foster City, CA, USA) with gene-specific primers and the results were normalized with β-actin as a control. PCR primers are listed in Additional file 1: Table S1.

Statistical analyses were performed using GraphPad Prism 7, and Student^’^s t-test and Mann-Whitney test were used to compare two groups of independent samples, as appropriate. ROC curve analysis was performed to evaluate the diagnostic value of circRNAs in CRC-m patients compared to CRC patients. Data were presented as mean ± SD, *p* < 0.05 was considered statistically significant.

## Prediction of circRNA-miRNA interactions

CircRNA-miRNA interaction were predicted by popular target prediction softwares, and network was constructed by Cytoscape software. Specific predictions based on miRanda, miRDB, miRWalk, RNA22 and Targetscan databases. And for each circRNA, we showed the top 5 miRNA that potentially bind to the circRNA and the five most likely target genes to every miRNAs.

## Findings

### Identification of differentially expressed circRNAs

**Fig. 1:**
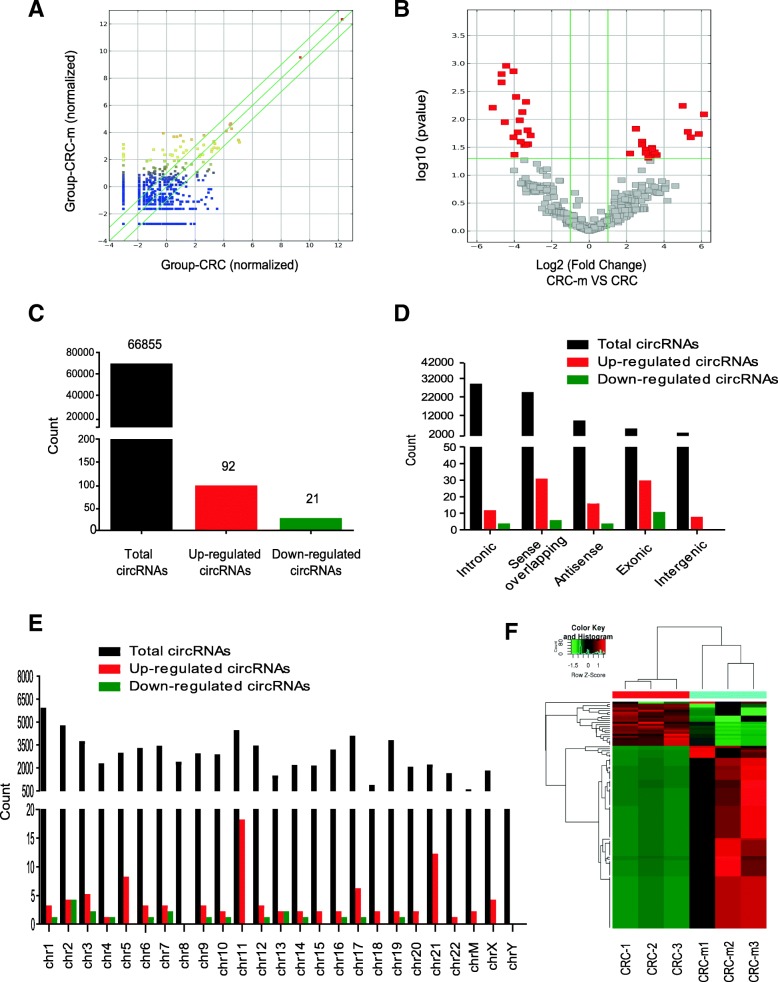
Analysis of differentially expressed circRNAs in the tissues from CRC patients with or without liver metastasis by RNA-sequencing. **a** The scatter plot figuratively expresses the changes in circRNA expression CircRNAs above the top green line and below the bottom green line demonstrated more than a 1.5-fold change between the two compared groups. **b** The volcano plot was showed the expression profiling between the two groups. The vertical green lines refer to a 2.0-fold (log2 scaled) up-regulation and down-regulation, respectively. The horizontal green line corresponds to a *P*-value of 0.05 (−log10 scaled). The red points in the plot represent differentially expressed circRNAs with statistical significance. **c** The amount of the total circRNAs and differentially expressed circRNAs. **d** CircRNAs were classified by category. **e** CircRNAs were distributed by located in human chromosomes. **f** Hierarchical clustering indicates differences in circRNA expression profiling between the two groups

To identify circRNAs that were differentially expressed in CRC patients with liver metastasis, the secondary sequencing was used to profile circRNAs expression in the tissue samples from three CRC patients with liver metastasis and three matched CRC patients. The basic characteristics of the patients was provided in Additional file 1: Table S2. Under the sequencing, total 66,855 circRNAs were detected in the tissue samples. The list of total circRNAs expression profiling was shown in Additional file 1: Table S3. The scatter plot showed the variation of circRNAs expression level between CRC patients with or without liver metastasis (Fig. 1a). As was showen in volcano plot, the significant differentially expressed circRNAs between the two groups were identified with fold-changes of greater than 2.0 and *p* value less than 0.05 (Fig. 1b). From the total 66,855 circRNAs, 92 circRNAs were significant up-regulated and 21 circRNAs were significant down-regulated (Fig. 1c and Additional file 1: Table S4). According to the source of the circRNA formation, it revealed that most significant differentially expressed circRNAs were transcribed from the exons of coding protein (Fig. 1d). Further, we classified of all differentially expressed circRNAs base on the different location on the chromosomes. All chromosomes have the expression of circRNA, and we could found the differentially expressed circRNAs except Y chromosome (Fig. 1e). Hierarchical clustering demonstrates these significant differentially expressed circRNAs (Fig. 1f). Thereinto, the top 10 circRNAs with significant up-regulation or down-regulation were listed in Additional file 1: Table S5, of these 20 circRNAs, the fold-changes were at least greater than 10. The circRNA-seq data and analysis suggest that some cricRNAs expressed level were different in the CRC patients with liver metastasis compared with CRC patients.

### Validation of differentially expressed circRNAs

**Fig. 2:**
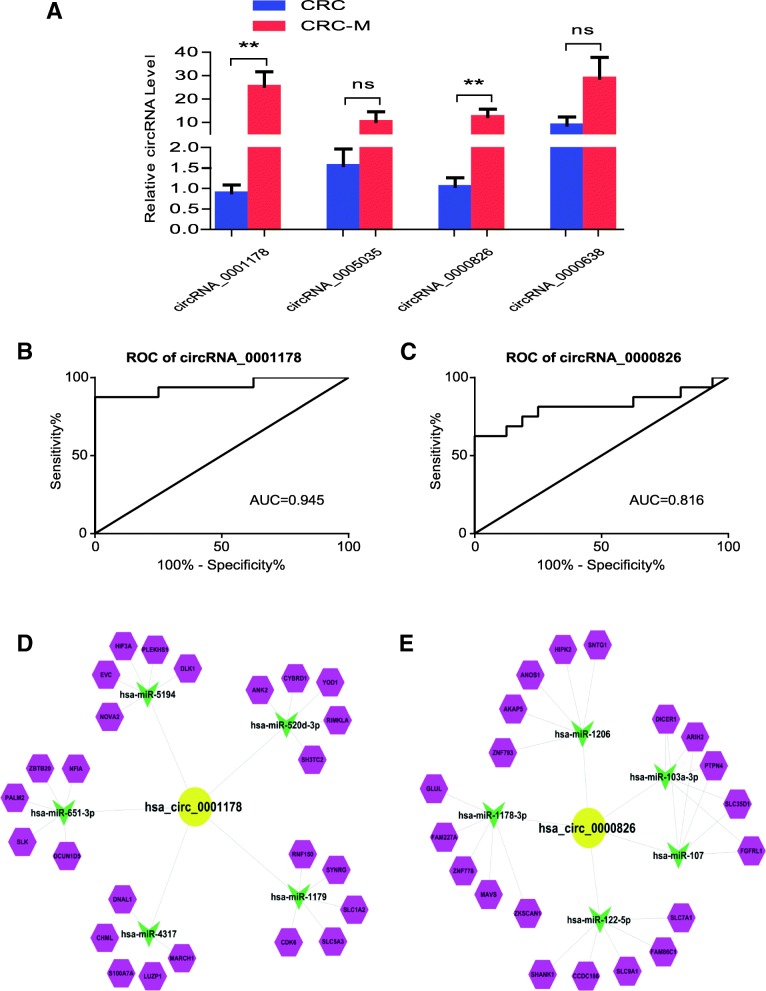
**a** Validation of the circRNAs expression by qRT-PCR. The expressions of top four up-regulated circRNAs in CRC-m samples from RNA-Seq data were evaluated using qRT-PCR in the sample from 32 CRC patients with or without liver metastasis. **b**, **c** ROC curve analysis of differentially expressed circRNAs. ROC curve analysis of circRNA_0001178 and circRNA_0000826 in CRC-m patients versus CRC patients. AUC values are given on the graphs. **d**, **e** ceRNA analysis for circRNA_0001178 and circRNA_0000826 respectively. The cytoscape software was used to show the circRNA-miRNA-mRNA interactions. Base on the miRNA prediction and bioinformatics analyses, we show the top 5 miRNAs may regulated by circRNA_0001178 (**d**), circRNA_0000826 (**e**) and top 5 target genes of each miRNA respectively

Considering the expression level of down-regulated circRNAs in CRC-m patients were very low and not easy to detect in disease progression, we only focus on the up-regulated circRNAs. From the list in Additional file 1: Table S5, we first selected the 4 up-regulated circRNAs which had the most significantly different expression. To verify the circRNA-seq results of these 4 circRNAs, qRT-PCR was performed in the tissue samples from 16 CRC-m patients and 24 CRC patients. As shown in Fig. 2a, the circRNA_0001178 and circRNA_0000826 were significantly up-regulated in CRC-m patients, which were consistent with previous sequencing results.

### ROC curve analysis of circRNA_0001178 and circRNA_0000826 in the CRC-m patients with liver metastasis

From the validation, we confirmed the expression levels of circRNA_0001178 and circRNA_0000826 were increased in CRC-m patients. Then the ROC curve analysis was performed to assess the diagnostic value of these two circRNAs in CRC-m patients. The AUC was 0.945 (95% CI: 0.863–1.000 *P* < 0.001) for circRNA_0001178 (Fig. 2b) and 0.816 (95% CI: 0.656–0.977, *P* < 0.01) for circRNA_0000826 (Fig. 2c). The results suggested the potential diagnostic value for these two circRNAs in CRC-m patients.

### circRNA-miRNA-mRNA network prediction and analyses

We built a circRNA-miRNA-target gene network for circRNA_0001178 and circRNA_0000826 using Cytoscape respectively. In the network map, we showed the top 5 miRNA that potentially bind to the circRNA and the five most likely target genes to each miRNA (Fig. 2d and e). From this figure, we can clearly see the potential target of circRNA_0001178 and circRNA_0000826. It gave us a clear direction to study the specific mechanism of these two circRNA in the liver metastasis of colorectal cancer.

## Conclusions

In this study, through high-throughput sequencing of patient tissues and bioinformatics analysis, we obtained the differential expression of circRNAs between the tissue samples from colorectal cancer patients with and without liver metastasis. And also indicated circRNA_0001178 and circRNA_0000826 were the promising biomarkers for liver metastases from colorectal cancer.

## Additional file

Additional file 1: **Table S1.** Sequences of the primers in this study. **Table S2.** Personal characteristic of patients participated in the sequencing. **Table S3.** CircRNA Expression Profiling in this study (please see the attached excel spreadsheet).**Table S4.** Differentially Expressed circRNAs between the two groups (please see the attached excel spreadsheet).**Table S5**. The Top 10 circRNAs with the significant upregulation or downregulation. (DOCX 19 kb)

## Abbreviations

CDK6: Cell division protein kinase 6
circRNAs: Circular RNAs
circRNA-Seq: Circular RNA sequencing
CRC: Colorectal cancer
CRC-m: Colorectal cancer liver metastases
