# Supplementary material for: RNA-Seq profiling of circular RNAs in human colorectal Cancer liver metastasis and the potential biomarkers
Source: Mol Cancer. 2019 Jan 10;18:8. doi: 10.1186/s12943-018-0932-8 (PMC6327571; doi:10.1186/s12943-018-0932-8)
Supplement: Supplementary file 1 — Table S1. Sequences of the primers in this study. Table S2. Personal characteristic of patients participated in the sequencing. Table S3. CircRNA Expression Profiling in this study (please see the attached excel spreadsheet).Table S4. Differentially Expressed circRNAs between the two groups (please see the attached excel spreadsheet).Table S5. The Top 10 circRNAs with the significant upregulation or downregulation. (DOCX 19 kb) [file 12943_2018_932_MOESM1_ESM.docx]

**Supplementary materials**

Table S1： Sequences of the primers in this study

| **Gene name** | **Forward primer (5’-3’)** | **Reverse primer (5’-3’)** |
| --- | --- | --- |
| hsa_circ_0001178 | GTGTGGGAAGCCAAGCAG | GGGTGTCATTAATCCCCG |
| hsa_circ_0005035 | GTGGGGACCTGTGTCCAG | GAAGCGGTAGCTGCGGTA |
| hsa_circ_0000826 | GGAGCGTCCAGTGGATGTA | GAACCCAGATTTGGGCATT |
| hsa_circ_0000638 | ATGCTGGCTTTGTTCCCA | TTCACCTCCAAGGCGTGT |
| β-actin | GTGGCCGAGGACTTTGATTG | CCTGTAACAACGCATCTCATATT |

Table S2： Personal characteristic of patients participated in the sequencing

| Group | Sex | Age | Tumor  histogenesis | Tumor differentiation | TNM  stage | Metastasis | Previous history of CRC |
| --- | --- | --- | --- | --- | --- | --- | --- |
| CRC-m tissue | M | 64 | Adenocarcinoma | [Moderate or poor differentiation](http://dict.cnki.net/dict_result.aspx?searchword=%e4%b8%ad%e4%bd%8e%e5%88%86%e5%8c%96&tjType=sentence&style=&t=moderate+or+poor+differentiation) | T4N2M1 | Lymph node, Liver | None |
| CRC-m tissue | M | 69 | Adenocarcinoma | [Poor differentiation](http://dict.cnki.net/dict_result.aspx?searchword=%e4%b8%ad%e4%bd%8e%e5%88%86%e5%8c%96&tjType=sentence&style=&t=moderate+or+poor+differentiation) | T3N2M1 | Lymph node, Liver | None |
| CRC-m tissue | M | 64 | Adenocarcinoma | [Moderate](http://dict.cnki.net/dict_result.aspx?searchword=%e4%b8%ad%e4%bd%8e%e5%88%86%e5%8c%96&tjType=sentence&style=&t=moderate+or+poor+differentiation)  [differentiation](http://dict.cnki.net/dict_result.aspx?searchword=%e4%b8%ad%e4%bd%8e%e5%88%86%e5%8c%96&tjType=sentence&style=&t=moderate+or+poor+differentiation) | T2N1M1 | Lymph node, Liver | None |
| CRC tissue | M | 69 | Adenocarcinoma | [Moderate](http://dict.cnki.net/dict_result.aspx?searchword=%e4%b8%ad%e4%bd%8e%e5%88%86%e5%8c%96&tjType=sentence&style=&t=moderate+or+poor+differentiation)  [differentiation](http://dict.cnki.net/dict_result.aspx?searchword=%e4%b8%ad%e4%bd%8e%e5%88%86%e5%8c%96&tjType=sentence&style=&t=moderate+or+poor+differentiation) | T2N0M0 | None | None |
| CRC tissue | M | 63 | Adenocarcinoma | [Moderate](http://dict.cnki.net/dict_result.aspx?searchword=%e4%b8%ad%e4%bd%8e%e5%88%86%e5%8c%96&tjType=sentence&style=&t=moderate+or+poor+differentiation)  [differentiation](http://dict.cnki.net/dict_result.aspx?searchword=%e4%b8%ad%e4%bd%8e%e5%88%86%e5%8c%96&tjType=sentence&style=&t=moderate+or+poor+differentiation) | T2N0M0 | None | None |
| CRC tissue | M | 66 | Adenocarcinoma | [Poor differentiation](http://dict.cnki.net/dict_result.aspx?searchword=%e4%b8%ad%e4%bd%8e%e5%88%86%e5%8c%96&tjType=sentence&style=&t=moderate+or+poor+differentiation) | T4N0M0 | None | None |

Table S3： CircRNA Expression Profiling in this study (please see the attached excel spreadsheet)

Table S4： Differentially Expressed circRNAs between the two groups (please see the attached excel spreadsheet)

Table S5： The Top 10 circRNAs with the significant upregulation or downregulation

| CircRNA ID | CircBase ID | Gene symbol | Fold changes | *P*-value |
| --- | --- | --- | --- | --- |
| chr21:17135210-17138460+ | circRNA_0001178 | USP25 | 69.9126854 | 0.0081136495103 |
| chr15:99250791-99251336+ | circRNA_0005035 | IGF1R | 57.7116503 | 0.0182007980684 |
| chr18:9182380-9221997+ | circRNA_0000826 | ANKRD12 | 38.4744335 | 0.0166442036582 |
| chr15:76566753-76588078- | circRNA_0000638 | ETFA | 12.1186872 | 0.0433166010878 |
| chr21:9827139-9827328+ | NA | XLOC_013994 | 12.1186872 | 0.0433166010878 |
| chr11:65267101-65267389+ | NA | MALAT1 | 11.2499926 | 0.0388192533959 |
| chr11:65267887-65268107+ | NA | MALAT1 | 11.2499926 | 0.0388192533959 |
| chr17:57808782-57816308+ | circRNA_0006508 | VMP1 | 11.2499926 | 0.0388192533959 |
| chr21:9827137-9827326+ | NA | XLOC_013994 | 11.2499926 | 0.0388192533959 |
| chr21:9827329-9827534+ | NA | XLOC_013994 | 11.2499926 | 0.0388192533959 |
| chr19:49994751-49995003+ | NA | RPL13A | -35.7031141 | 0.0061638297637 |
| chr22:41568621-41569656+ | NA | EP300 | -25.6400092 | 0.0015435503252 |
| chr6:31238920-31324013- | NA | HLA-C | -25.6400092 | 0.0021675005632 |
| chr14:20811417-20811568+ | NA | RPPH1 | -22.7658668 | 0.0111947184945 |
| chr3:47139445-47144913- | circRNA_0001289 | SETD2 | -21.6256213 | 0.0010966052031 |
| chr13:21305980-21306260- | circRNA_0003285 | N6AMT2 | -16.5034378 | 0.0209006567705 |
| chr7:74171152-74172333+ | circRNA_0006672 | GTF2I | -16.5034378 | 0.0013719349149 |
| chr9:33971649-33973235- | circRNA_0001851 | UBAP2 | -15.933894 | 0.0428863424727 |
| chr4:2352883-2353441- | NA | ZFYVE28 | -14.9943683 | 0.0039503719283 |
| chr2:61343114-61345251+ | circRNA_0007793 | KIAA1841 | -14.1102408 | 0.0169577987367 |
